# Antifungal activity of volatile organic compounds from essential oils against the postharvest pathogens *Botrytis cinerea*, *Monilinia fructicola*, *Monilinia fructigena*, and *Monilinia laxa*

**DOI:** 10.3389/fpls.2023.1274770

**Published:** 2023-10-04

**Authors:** Samuel Álvarez-García, Marwa Moumni, Gianfranco Romanazzi

**Affiliations:** ^1^ Department of Agricultural, Food and Environmental Sciences, Marche Polytechnic University, Ancona, Italy; ^2^ Plant Physiology Area, Engineering and Agricultural Sciences Department, Universidad de León, León, Spain

**Keywords:** cinnamon, fumigation, lavender, oregano, rosemary, tea tree, thyme, VOC chamber

## Abstract

Gray mold and brown rot, caused respectively by *Botrytis cinerea* and *Monilinia* spp., are fungal diseases responsible for significant losses during the storage of fruit and vegetables. Nowadays, the control of postharvest diseases is shifting towards more sustainable strategies, including the use of plant secondary metabolites. In this study, the antifungal activity of *Origanum vulgare*, *Thymus vulgaris*, *Thymus serpyllum*, *Melaleuca alternifolia*, *Lavandula officinalis*, *Lavandula hybrida*, *Citrus bergamia*, *Rosmarinus officinalis*, *Cinnamomum zeylanicum* essential oils (EOs) in vapor phase was tested *in vitro* against *B. cinerea, Monilinia fructicola*, *Monilinia fructigena*, and *Monilinia laxa*. For the experiments, a protocol using a volatile organic compounds (VOC) chamber was designed. Results indicate a dose-dependent inhibitory activity of all the tested EOs, with *O. vulgare*, *T. vulgaris*, and *T. serpyllum* being the most active ones, with minimum inhibitory concentrations (MIC) of 22.73, 45.45, and 22.73 µl/L, respectively, against *B. cinerea* and a range between 5.64 and 22.73 µl/L against the three *Monilinia* spp. Overall, *B. cinerea* presented lower sensitivity to vapor-phase EOs than any of the *Monilinia* strains, except for the *C. zeylanicum* EO, which consistently showed higher inhibition against *B. cinerea*. Among the three *Monilinia* spp., *M. fructicola* was the least sensitive, while *M. fructigena* was the most sensitive. The use of VOC chambers proved to be a reliable protocol for the assessment of antimicrobial activities of EOs. These results suggest that the VOC emitted by the tested EOs are effective towards important decay-causing fungi, and that they could be used for the control of gray mold and brown rot in *in vivo* trials.

## Graphical Abstract

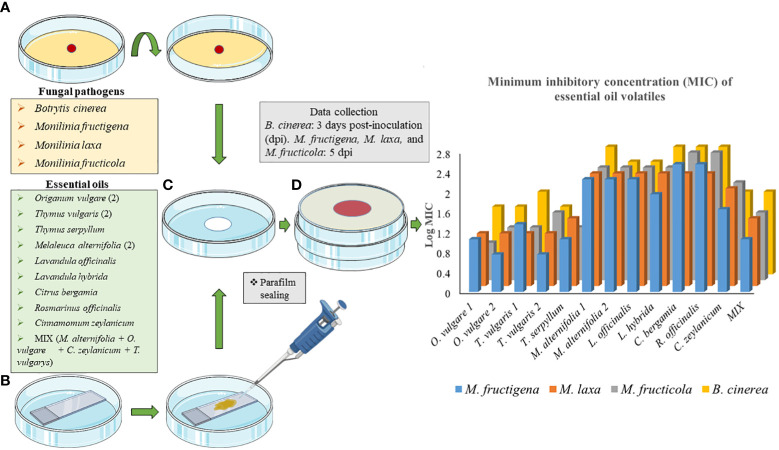

## 1 Introduction

Essential oils (EOs) are complex and diverse mixtures of chemical compounds, many of them of volatile nature, which can be obtained from plant organs and tissues through different extraction methods (El Khetabi et al., 2022). They present relevant biological activities, such as attraction or repellency, antioxidant, insecticidal, and antimicrobial effects. EOs have been proven to produce significant antimicrobial activity against many phytopathogenic fungi, both in direct liquid contact and in vapor phase (Reyes-Jurado et al., 2020; El Khetabi et al., 2021; Ma et al., 2022). This action depends on the specific sensitivity of each microbial strain and on several characteristics of the EOs, including their composition, the proportion of each individual compound, and their ability to diffuse and reach the microbial target (Pinto et al., 2020; El Khetabi et al., 2022).

EO composition vary not only according to the plant species, but also to the variety, cultivar, plant part, physiological state, and extraction method (Kloucek et al., 2012; El Khetabi et al., 2022; Reyes-Jurado et al., 2020). This overwhelming heterogeneity leads to an intricate overlapping of modes of action that can impair microbial growth. EOs affect different target structures in microorganisms, damaging cell membranes, the cell wall, genetic material, or membrane proteins (Ayala-Zavala et al., 2008; Usall et al., 2016), thus disrupting important physiological and metabolic processes leading to the inactivation of essential enzymes, leakage of cell content, depletion of proton motive active sites, spore disruption, or the induction of apoptotic pathways, among others (Usall et al., 2016; Spadaro et al., 2021). As a result, EOs and their individual compounds usually present additive or synergistic antimicrobial activity (Derbassi et al., 2022). On the contrary, a few studies have reported fungal growth promotion or an increase in disease incidence induced by some EOs in certain conditions (Santoro et al., 2018). Therefore, dosage needs to be carefully evaluated and controlled to avoid unwanted adverse effects in eventual commercial applications.

EO complexity leads to challenges in both the study and subsequent application of EO VOCs for microbial control. EO hydrophobicity and volatility compromise their effectivity in direct contact, affecting the availability and diffusion of the individual active compounds, and imposing the use of solvents or emulsifiers that may affect the resulting outcome (Kloucek et al., 2012). The testing of EO VOCs against postharvest pathogens relies mostly on *in vitro* methodologies derived from the protocol proposed by Maruzzella and Sicurella (1960), using an upside-down Petri dish, or the introduction of plates inside bigger containers (Schmidt et al., 2017; Song et al., 2019). In general, the study of volatile interactions traditionally faces methodological difficulties such as the leakage of active compounds, limitation of oxygen availability and gas exchange, determination of the real concentrations reached inside the experimental units, and a general lack of standardized materials and protocols (Kai and Piechulla, 2009; Kloucek et al., 2012).

Nevertheless, biological interactions mediated by volatile organic compounds (VOCs) from plants, fungi, yeasts and bacteria have been explored for decades (Dennis and Webster, 1971; Avalos et al., 2018; Tilocca et al., 2020). Although studies which have focused on the direct contact of EOs with the fungal strains via growth media, liquid mixtures, or coatings are still prevalent (Kapetanakou et al., 2019; Gonçalves et al., 2021), a great interest is arising towards the fumigant antifungal activity of EO volatiles, and for their potential use in postharvest treatment of fruits and vegetables (Cindi et al., 2015; Taghavi et al., 2018; Reyes-Jurado et al., 2020; Ma et al., 2022).

This control of postharvest spoilage is of the utmost importance, as it is estimated that nearly half of all fruits and vegetables produced on the planet are lost or not consumed (FAO, 2021). Plant diseases are responsible for significant losses, which annually cost the global economy over $220 billion. Gray mold and brown rot, caused respectively by *Botrytis cinerea* Pers. and *Monilinia* spp. can be counted among some of the most destructive and widespread postharvest fungal diseases (Santoro et al., 2018; Roca-Couso et al., 2021; De Miccolis Angelini et al., 2022).

*Botrytis cinerea* is a ubiquitous phytopathogen that affects a vast range of agricultural plants as well as causing postharvest decay of fruits and vegetables. This pathogen can occur in the field and remain in a latent state up until later phases of postharvest management, where it emerges as a pathogen (Powelson, 1960). At this stage, the infection can quickly spread to neighboring fruits in a process known as nesting, with the subsequent increase in product loss. Moreover, this fungus can grow at low temperatures, further compromising the storage and marketing stages (Roca-Couso et al., 2021). *Monilinia fructicola* (G. Winter) Honey, *Monilinia fructigena* (Pers.) Honey, and *Monilinia laxa* (Aderh. and Ruhland) Honey are among the most common postharvest fungal pathogens affecting stone and pome fruits, and are the causal agents of brown rot and blossom blight on fruit trees, resulting in important yield losses, postharvest fruit waste, and reduced shelf life (Santoro et al., 2018; Casals et al., 2022; De Miccolis Angelini et al., 2022).

Nowadays, the focus in the control of postharvest diseases is shifting towards more sustainable strategies, including the use of biological control agents and natural compounds such as plant and microbial extracts and secondary metabolites (Romanazzi et al., 2022). Among them, plant essential oils (EOs) have been proven to mediate diverse biological interactions, standing out their significant antimicrobial activity against many phytopathogenic fungi, both in direct liquid contact and in vapor phase (Reyes-Jurado et al., 2020; El Khetabi et al., 2021; Moumni et al., 2021). Numerous plant EOs in volatile phase present antifungal effects against postharvest pathogens, including *B. cinerea* (Servili et al., 2017; Pinto et al., 2020; Di Francesco et al., 2022) and *Monilinia* spp. (Spadaro et al., 2021; Santoro et al., 2018; Khumalo et al., 2017).The strong antifungal effect of vapor-phase EOs from *T. vulgaris*, *C. zeylanicum*, *O. vulgare* and other *Origanum* species on *B. cinerea* has been extensively reported (Munhuweyi et al., 2017; Zhao et al., 2021; Elsayed et al., 2022; Kaharamanoglu et al., 2022; Kara et al., 2022). Inhibitory effects of some EOs volatiles against *M. fructicola* have been also reported, such as *T. vulgaris* (Sellamuthu et al., 2013; Spadaro et al., 2021) and *M. alternifolia* (Xu et al., 2022). *O. vulgare* (Carović-Stanco et al., 2013) and *T. vulgaris* (Khumalo et al., 2017; Pinto et al., 2020) EO VOCs also inhibited *M. laxa* growth. Nevertheless, there are many plant EOs that have not been tested yet in volatile phase against these major phytopathogenic fungi. This is especially true for *M. fructicola*, *M. laxa*, and *M. fructigena*, for which very few EO volatiles have been assayed to date. Therefore, there is a need to further explore the antifungal activity of EOs in volatile phase against these postharvest pathogens in order to develop alternative strategies for their control.

The main objectives of the present work were: (i) to develop an effective protocol for the evaluation of these interactions using VOC chambers; and (ii) to evaluate and compare the *in vitro* antifungal activity of the VOCs released by twelve EOs and a commercial mixture against the postharvest pathogens *B. cinerea*, *M. fructicola*, *M. fructigena*, and *M. laxa*.

## 2 Materials and methods

### 2.1 Microbial strains and culture conditions

Four postharvest pathogenic fungi were evaluated in the present work for their susceptibility to EOs in vapor phase: *B. cinerea* (strain B05.10; Netherlands), and *M. fructicola*, *M. fructigena*, and *M. laxa* isolated from infected nectarines in Italy (collection of Plant Pathology Unit, Marche Polytechnic University, Italy). These strains had been previously identified in our laboratory by morphological and molecular methods using multiplex PCR (Makau et al., 2023) following the method proposed by Côté et al. (2004). They were stored at 4 °C and refreshed by growing them on potato dextrose agar (PDA; 40 g/L; Scharlab S.L., Sentmenat, Spain) at 22 °C ± 2 °C prior to their use in the experiments.

### 2.2 Essential oils

Twelve commercial essential oils were tested individually to assess their vapor-phase antifungal activity against the referred fungal strains: *Origanum vulgare*, *Thymus vulgaris*, *Thymus serpyllum*, *Melaleuca alternifolia*, *Lavandula officinalis*, *Lavandula hybrida*, *Citrus bergamia*, *Rosmarinus officinalis*, and *Cinnamomum zeylanicum*. EOs from two different providers were tested in the case of *O. vulgare*, *T. vulgaris*, and *M. alternifolia*. A commercial mixture (MIX) composed of 25% *M. alternifolia*, 25% *O. vulgare*, 25% *C. zeylanicum*, and 25% *T. vulgaris* was also assayed. The EOs, including the mixture, were kindly provided by Flora srl (Pisa, Italy) and GreenVet (Forli, Italy). The extraction method, plant material, and purity grade of the tested EOs (100% pure) were certified by the suppliers (**Table 1** **)**.

**Table 1:** Essential oils information regarding origin, year and method of extraction, and main components.

Plant	Common name	Plant part	Provider	Main components
*Origanum vulgare* 1	Oregano	whole plant in flower	Flora srl, Pisa (IT)	carvacrol 69.63%, ɣ-terpinene 6.69%, p-cymene 5.11%, thymol 4.82%, β-myrcene 2.12%
*Origanum vulgare* 2	Oregano	whole plant in flower	GreenVet, Forlì (IT)	carvacrol 79.29%, ɣ-terpinene 8.85%, p-cymene 6.67%, thymol 2.98%, sabinene 0.60%, limonene 0.13%, myrcene 0.11%, cis-sabinene hydrate 0.11%
*Thymus* vulgaris 1	Red thyme	whole plant in flower	Flora srl	p-cymene 31.9%, thymol 24.2%, 1,8-cineol 8.07%, limonene 7.04%, ɣ-terpineol 6.12%, t-caryophyllene 3.97%, carvacrol 3.44%, camphene 2.49%, α-pinene 2.14%, borneol 2.09%, β-phellandrene 1.89%, linalool 1.72%, ɣ-terpinene 1.11%, phellandrene 0.63%, α-thujene 0.18%, β-myrcene 0.13%, 3-octanol 0.13%, 4-terpineol 0.07%, carvacrol methyl ether 0.05%, camphor 0.02%, germacrene 0.02%, caryophyllene oxide 0.02%, t-sabinene hydrate 0.01%
*Thymus* vulgaris 2	Red thyme	whole plant in flower	GreenVet	thymol 46.41%, p-cymene 19.89%, ɣ-terpinene 9.47%, carvacrol 6.17%, linalool 3.71%, β-caryophyllene 2.71%, α-pinene 1.21%, limonene 1.12%, camphene 1.08%, α-terpinene 1.02%, terpinen-4-ol 0.90%, geranial 0.89%, α-thujene 0.80%, myrcene 0.69%, α-terpineol 0.55%, α-phellandrene 0.32%, α-humulene 0.25%, 1,8-cineol 0.20%, caryophyllene oxide 0.19%, p-cymen-8-ol 0.16%, sabinene 0.14%, terpinolene 0.13%, myrtenal 0.11%
*Thymus serpyllum*	White thyme	whole plant in flower	Flora srl.	carvacrol 54.69%, linalool 25.91%, p-cymene 3.69%, ɣ-terpinene 3.42%, thymol 1.92%
*Melaleuca alternifolia* 1	Tea tree	leaves	Flora srl.	terpinene 4-ol 39.01%, ɣ-terpinene 20.58%, α-terpinene 10.35%, terpinolene 3.91%, a-terpineil 2.69%
*Melaleuca alternifolia* 2	Tea tree	leaves	GreenVet	terpinene 4-ol 41.21%, ɣ-terpinene 17.69%, α-terpinene 8.31%, α-terpineol 6.55%, α-pinene 4.53%, carvacrol 3.35%, p-cymene 3.02%, terpinolene 2.60%, 1,8-cineole 2.56%, limonene 2.55%, aromadendrene 2.05%, viridiflorene 1.19%, caryophyllene oxide 0.48%, cis-linalool oxide 0.34%, camphor 0.29%, valencene 0.26%, α-gurjunene 0.24%, β-pinene 0.21%, β-salinene 0.19%, α-phellandrene 0.17%, sabinene 0.16%, myrtenal 0.15%, lavandulyl acetate 0.13%, trans-ocimene 0.10%
*Lavandula officinalis*	Lavender	flowers	Flora srl.	linalool 31.30%, linalyl acetate 30.93%, β-caryophyllene 4.87%, β-farnesene 3.95%, c-β-ocimene 3.57%
*Lavandula hybrida*	Hybrid lavender; lavandin	flowers	Flora srl.	linalyl acetate 33.5%, linalool 29.2%, camphor 8.3%, 1,8-cineol 6.2%, 4-terpineol 3.37%
*Citrus bergamia*	Bergamot	peel	Flora srl.	limonene 40.50%, linalyl acetate 27.17%, linalool 11.59%, ɣ-terpinene 7.17%, β-pinene 5.84%
*Rosmarinus officinalis*	Rosemary	whole plant in flower	Flora srl.	camphor 20.5%, 1,8-cineol 20.2%, α-pinene 18.6%, camphene 8.35%, limonene 5.71%
*Cinnamomum zeylanicum*	Cinnamon	inner bark	GreenVet	trans-cinnamaldehyde 79.03%, β-caryophyllene 5.63%, 1,8-cineol 3.97%, citronellyl acetate 2.60%, trans-cinnamyl acetate 1.49%, a-humulene 0.69%, limonene 0.67%, carvacrol 0.56%, caryophyllene oxide 0.22%, trans-rose oxide 0.21%, pulegone 0.19%, cis-sabinene hydrate 0.12%, piperitone 0.12%
(MISCELA AN-50G)	MIX	–	GreenVet	25% *M. alternifolia*; 25% *O. vulgare*; 25% *C. zeylanicum*; 25% *T. vulgaris*

All details were provided by the manufacturers.

### 2.3 *In vitro* antifungal activity of essential oils in vapor phase using VOC chambers

**Figure 1:**
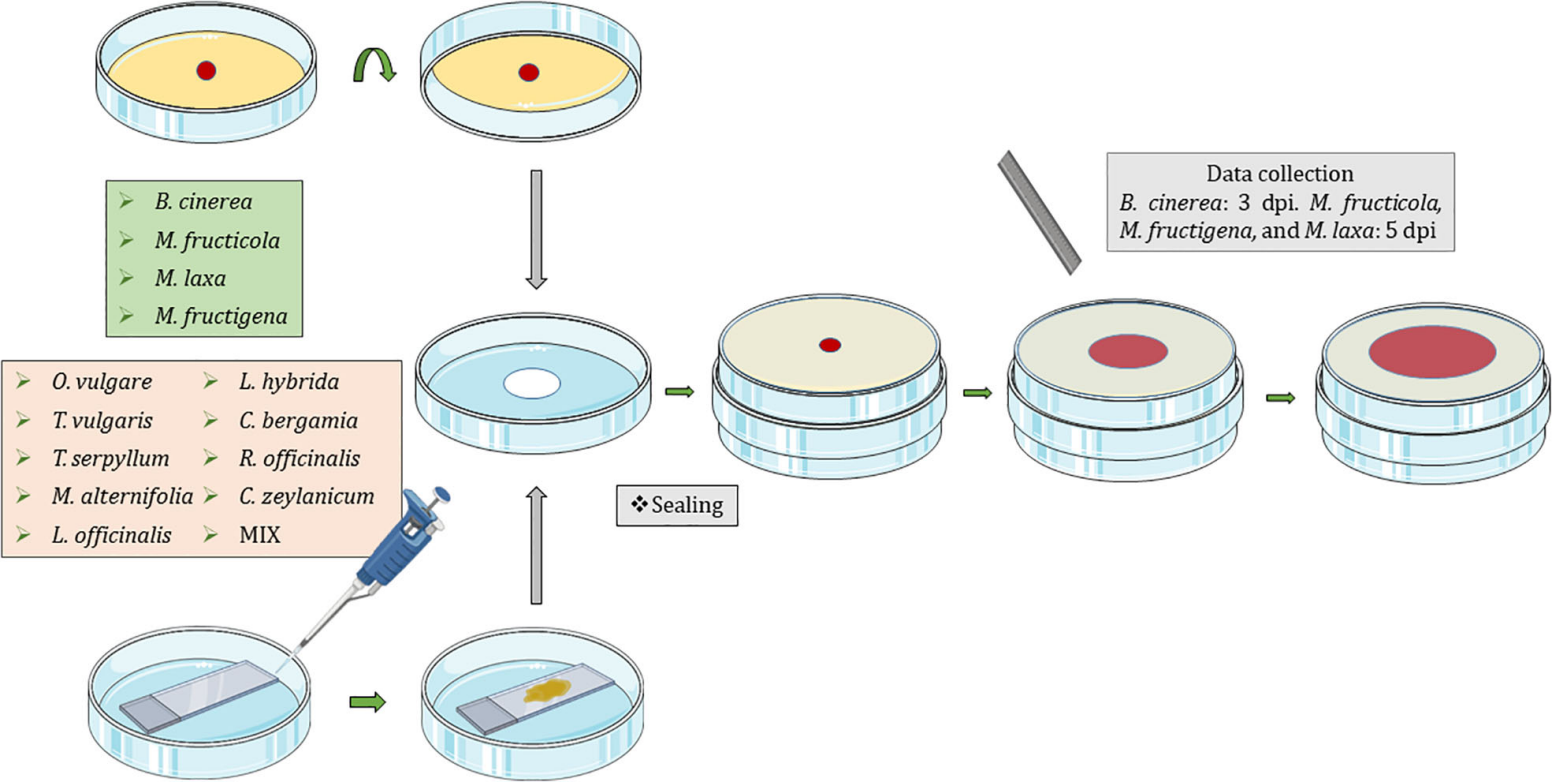
Graphical representation of the new protocol using non-vented VOC chambers (Álvarez-García et al., 2021) for the exposure of *Botrytis cinerea*, *Monilinia fructicola*, *Monilinia fructigena*, and *Monilinia laxa* to the VOCs released by *Origanum vulgare*, *Thymus vulgaris*, *Thymus serpyllum*, *Melaleuca alternifolia*, *Lavandula officinalis*, *Lavandula hybrida*, *Citrus bergamia*, *Rosmarinus officinalis*, and *Cinnamomum zeylanicum* EOs; and a mixture (MIX) of 25% *M. alternifolia*, 25% *O. vulgare*, 25% *C. zeylanicum*, and 25% *T. vulgaris*. Dpi, days post inoculation.

**Figure 2:**
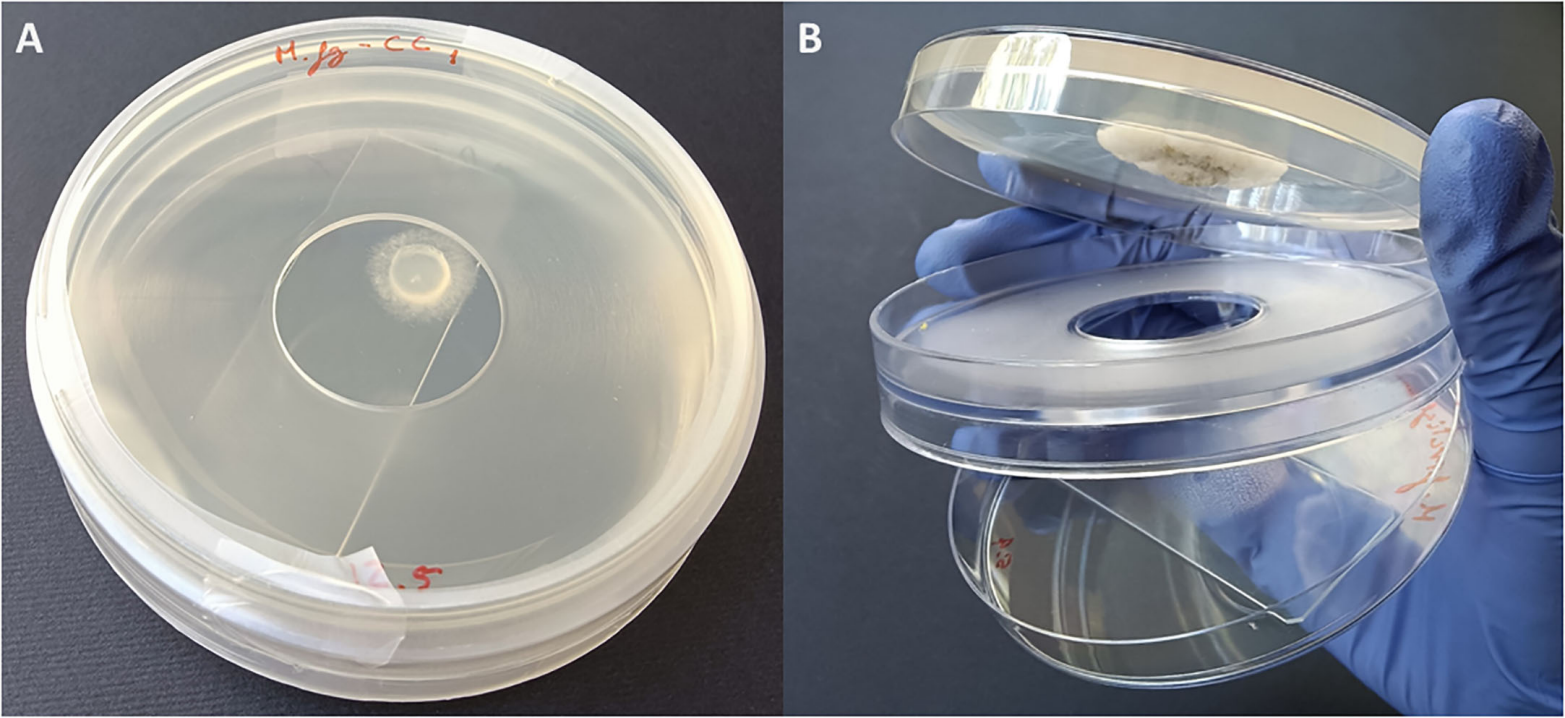
**(A)** Closed VOC Chamber with EO spread on a glass slide in the lower plate, upper plate containing the fungal plug cultured on 20 mL PDA medium, and a central piece holding both plates together and sealed with parafilm. **(B)** Open VOC Chamber showing the glass slide with EO in the lower plate and a fungal colony growing in the upper one. The hole that allows the flow of VOCs between plates can be clearly seen in the central piece.

Fungi were exposed to the volatiles of the listed EOs (**Table 1**) using non-vented VOC chambers (Álvarez-García et al., 2021) (JD. Catalán S.L., Madrid; Spain. Produced specifically by request for these experiments). An adequate dose of pure EO was placed on top of a microscopy glass slide (Thermo Scientific, Gerhard Menzel GmbH, Braunschweig, Germany) inside a Petri dish. This plate formed the lower base of the VOC Chamber, while its lid was substituted by a VOC chamber central piece (**Figures 1**, **2**). On the other side, an 8 mm diameter plug of the corresponding fungal strain was inoculated onto the center of another Petri dish containing 20 mL of PDA. These Petri dishes were flipped over and placed upside down on top of the central piece, forming the upper plate of the fully assembled VOC chamber (**Figures 1**, **2**). In this way, the lower plate containing the EO and the upper plate with the fungi are held together facing each other and connected by the hole in the central piece, allowing the free flow of VOCs from one compartment to the other (**Figures 1**, **2**). The chambers were sealed with two layers of Parafilm (Amcor-Bemis, USA) and put into a thermostatic cabinet (Lovibond TC255S, Tintometer GmbH, Germany) at 22 °C ± 2 °C.

From three up to eight doses were assessed depending on the specific EO, having been selected considering the bibliography and the sensitivity of each fungal strain in accordance with preliminary assays. Concentrations are expressed as microliters of EO per liter of air inside the VOC chamber (µL/L) by calculating the air volume of the headspace (volume of the two 90mm Petri dishes forming the chamber minus 20 ml corresponding to the PDA, giving a final volume of 0.11 L of air per VOC chamber). Lowest concentrations were set up at 2.82 µL/L for all EOs, while the highest ones were determined by assessing the inhibitory activity of each individual treatment. The EO volumes were measured using a micropipette (DLAB Scientific Co., ltd., China). The specific concentrations tested for each EO and fungal strain can be found in the results section alongside their corresponding percentages of inhibition (PIs, %). Inoculated plates without any EO were used as controls. Three replicates were performed per treatment in a single assay. PIs were determined using the following equation: PI = [(C - T)/C] x 100 (Gotor-Vila et al., 2017). Where C is the diameter of the control colony and T that of the treatment, both after subtracting 8 mm corresponding to the diameter of the inoculation plug, as proposed by Mutawila et al. (2016).

Growth was assessed by measuring two perpendicular diameters of each replicate, considering the edge of the colony. Data were collected 3 days post inoculation (dpi) for *B. cinerea*; and 5 dpi for *M. fructicola*, *M. fructigena*, and *M. laxa*, when mycelial growth of one of the treatments reached the edge of the plate, and PIs were subsequently calculated in comparison to the growth of the untreated control. The Minimum Inhibitory Concentration (MIC) for each EO and fungal strain was determined as the lowest tested concentration that showed no mycelial growth inhibition at the last day of the experiment (Fontana et al., 2021). A rank analysis (Conover and Iman, 1981) was performed considering the MIC for each fungus and treatment.

The inoculation plugs from those treatments presenting complete inhibition were re-inoculated on fresh PDA to evaluate whether the activity of the volatile EOs was fungicidal or fungistatic (Moumni et al., 2021), and eventual growth was recorded 5 dpi.

### 2.4 Data treatment and statistical analyses

After testing the data normality and equality of variances with Kolmogorov-Smirnov´s test and Levene´s test, statistical analyses were carried out using one-way analysis of variance (ANOVA), and means were separated with Tukey’s *post hoc* test (p ≤ 0.05). A non-parametric Kruskal–Wallis H-test was used, and means were separated with the Mann-Whitney U-test (p ≤ 0.05) when data were not normally distributed or homoscedastic. All statistical analyses were performed using IBM SPSS Statistics 26 (Armonk, NY, United States).

## 3 Results

### 3.1 Minimum inhibitory concentrations of essential oils in the vapor phase against postharvest fungal pathogens

Results indicate a dose-dependent inhibitory activity of VOCs from all tested EOs against the fungal strains. Overall, they exerted lower antifungal effects against *B. cinerea* in comparison to all three *Monilinia* spp. (**Table 2**). The rank analysis showed the following order of sensitivity: *B. cinerea* < *M. fructicola* < *M. laxa* < *M. fructigena*. *O.* vulgare, *T. serpyllum* and *T. vulgaris* showed lower MICs, ranging from 22.73 to 45.45 μL/L for *B. cinerea* and from 5.54 to 22.73 μL/L against the different *Monilinia* spp. strains. *C. zeylanicum* rendered moderate results, with MICs of 45.45 μL/L against *B. cinerea* and *M. fructigena*, and of 90.91 μL/L for *M. fructicola* and *M. laxa*. This was the only treatment in which some of the *Monilinia* spp., specifically *M. fructicola* and *M. laxa*, presented higher MIC than *B. cinerea*. *M. alternifolia*, *L. officinalis*, and *L. hybrida* EOs presented higher MICs, being 181.82 to 363.64 μL/L against *B. cinerea* and 90.91 to 181.82 μL/L for *Monilinia* spp., while those of *C. bergamia* and *R. officinalis* presented an MIC of 363.64 μL/L against *B. cinerea*, *M. fructicola*, and *M. fructigena*, and of 181.82 μL/L for *M. laxa*. The commercial mixture (MIX) exerted a considerable inhibition, mostly in the range of the two tested *Thymus* spp., with MICs: 45.45 μL/L against *B. cinerea*, 22.73 μL/L for *M. fructicola* and *M. laxa*, and 11.76 μL/L against *M. fructigena*. Some MIC differences were also observed between EOs derived from the same plant but from a different provider (*O. vulgare*, *T. vulgaris*, *M. alternifolia*). MICs of all the tested EOs against the four fungal strains are presented in **Table 2**.

**Table 2:** Minimum Inhibitory Concentration (MIC) of VOCs from twelve individual essential oils and a commercial mixture against *Botrytis cinerea* (3 dpi), *Monilinia fructicola*, *Monilinia fructigena*, and *Monilinia laxa* (5 dpi).

Essential oil	MIC (μL/L of air)			
	*B. cinerea*	*M. fructicola*	*M. fructigena*	*M. laxa*
*Origanum vulgare* 1	22.73	5.64	11.36	11.36
*Origanum vulgare* 2	22.73	11.36	5.64	11.36
*Thymus vulgaris* 1	45.45	11.36	22.73	11.36
*Thymus vulgaris* 2	22.73	22.73	5.64	11.36
*Thymus serpyllum*	22.73	11.36	11.36	22.73
*Melaleuca alternifolia* 1	363.64	181.82	181.82	181.82
*Melaleuca alternifolia* 2	181.82	181.82	181.82	181.82
*Lavandula officinalis*	181.82	181.82	181.82	181.82
*Lavandula hybrida*	363.64	181.82	90.91	181,82
*Citrus bergamia*	363.64	363.64	363.64	181.82
*Rosmarinus officinalis*	363.64	363.64	363.64	181.82
*Cinnamomum zeylanicum*	45.45	90.91	45.45	90.91
MIX*	45.45	22.73	11.36	22.73

* 25% M. alternifolia, 25% O. vulgare, 25% C. zeylanicum, 25% T. vulgaris.

### 3.2 Inhibition by essential oils in the vapor phase against postharvest fungal pathogens

The antifungal effects of all tested EOs were demonstrated to be dose dependent, with percentage of inhibition (PI) rising as concentrations increased (**Tables 3**–**6**).

**Table 3:** Percentage of Inhibition (PI, %) produced by the tested EOs in vapor phase against *Botrytis cinerea*.

Essential oil	PI (%) exerted by EO-VOCs on *Botrytis cinerea* mycelial growth 3 days post inoculation. EO concentrations are expressed in μL/L							
	2.82	5.64	11.36	22.73	45.45	90.91	181.82	363.64
*O. vulgare* 1	67.6 ± 2.0 d, AB	82.9 ± 2.1 c, ABC	94.4 ± 1.9 b, AB	100 a, A	100 a, A	–	–	–
*O. vulgare* 2	87.0 ± 3.7 b, A	89.1 ± 2.2 b, AB	97.5 ± 0.6 a, A	100 a, A	100 a, A	–	–	–
*T. vulgaris* 1	50.9 ± 2.8 d, BC	71.8 ± 3.8 c, C	86.3 ± 4.2 b, B	97.9 ± 0.7 a, AB	100 a, A	100 a, A	–	–
*T. vulgaris* 2	91.9 ± 0.6 d, A	93.7 ± 1.1 c, A	95.4 ± 0.6 b, A	100 a, A	100 a, A	100 a, A	–	–
*T. serpyllum*	66.9 ± 2.2 d, AB	78.5 ± 5.5 c, BC	90.5 ± 1.1 b, AB	100 a, A	100 a, A	100 a, A	–	–
*M. alternifolia* 1	21.1 ± 1.1 d, D	19.7 ± 1.1 d, EF	16.0 ± 5.9 d, FG	23.6 ± 3.9 d, E	43.5 ± 1.7 c, E	73.6 ± 4.4 b, D	94.6 ± 1.4 a, B	100 a
*M. alternifolia* 2	29.6 ± 23.3 d, CD	48.2 ± 13.9 cd, D	55.6 ± 2.1 cd, D	66.9 ± 9.8 bc, C	85.9 ± 1.2 ab, B	95.4 ± 0.6 ab, AB	100 a, A	100 a
*L. officinalis*	18.7 ± 3.0 e, D	16.7 ± 5.6 e, EF	16.0 ± 0.7 e, FG	36.8 ± 3.9 d, D	60.6 ± 2.8 c, D	90.2 ± 0.9 b, BC	100 a, A	100 a
*L. hybrida*	24.5 ± 0.4 e, D	27.3 ± 1.7 e, EF	23.1 ± 4.6 e, F	36.8 ± 2.5 d, D	69.9 ± 1.1 c, C	90.0 ± 3.1 b, BC	99.8 ± 0.4 a, A	100 a
*C. bergamia*	24.1 ± 2.8 e, D	28.9 ± 0.4 de, E	35.2 ± 2.9 cd, E	40.5 ± 1.4 c, D	45.1 ± 6.7 c, E	84.0 ± 6.7 b, C	96.1 ± 0.4 a, B	100 a
*R. officinalis*	15.0 ± 0.4 e, D	13.2 ± 4.2 e, F	11.3 ± 0.8 de, G	14.8 ± 0.8 de, E	20.8 ± 1.4 d, F	41.3 ± 2.4 c, E	78.4 ± 3.5 b, C	100 a
*C. zeylanicum*	37.9 ± 19.2 c, CD	43.9 ± 2.0 c, D	69.9 ± 0.4 b, C	89.6 ± 3.6 ab, B	100 a, A	–	–	–
MIX	78.8 ± 4.1 c, A	86.6 ± 2.3 b, AB	91.6 ± 1.7 b, AB	98.7 ± 1.1 a, AB	100 a, A	–	–	–

The results are expressed as the mean ± standard deviation, r=3. The statistical analyses were carried out using one-way analysis of variance (ANOVA), followed by Tukey’s post hoc test (p ≤ 0.05) or non-parametric Kruskal–Wallis H-test, followed by Mann-Whitney U-test (p ≤ 0.05). Different lower-case letters indicate statistical differences between concentrations of the same EO (horizontal rows). Different upper-case letters indicate statistical differences between the same concentration of different EOs (vertical columns).

**Table 4:** Percentage of Inhibition (PI, %) produced by the tested EOs in vapor phase against *Monilinia fructicola*.

Essential Oil	PI (%) exerted by EO-VOCs on *Monilinia fructicola* mycelial growth 5 days post inoculation. EO concentrations are expressed in μL/L							
	2.82	5.64	11.36	22.73	45.45	90.91	181.82	363.64
*O. vulgare* 1	62.0 ± 24.0 b, BC	100 a, A	100 a, A	–	–	–	–	–
*O. vulgare* 2	90.0 ± 1.6 c, A	96.3 ± 1.4 b, AB	100 a, A	–	–	–	–	–
*T. vulgaris* 1	62.5 ± 10.0 b, BC	89.3 ± 3.0 a, AB	100 a, A	–	–	–	–	–
*T. vulgaris* 2	84.6 ± 7.3 b, AB	95.0 ± 1.0 a, AB	98.9 ± 0.4 a, A	100 a, A	–	–	–	–
*T. serpyllum*	72.0 ± 1.7 c, ABC	84.2 ± 6.1 b, B	100 a, A	–	–	–	–	–
*M. alternifolia* 1	8.9 ± 1.8 g, DE	31.5 ± 3.9 f, DE	42.2 ± 2.2 e, B	57.3 ± 1.2 d, BC	65.0 ± 0.7 c, C	83.0 ± 0.8 b, C	100 a, A	–
*M. alternifolia* 2	29.7 ± 3.2 e, D	33.5 ± 1.0 e, D	46.3 ± 2.0 d, B	53.0 ± 4.8 c, BC	64.3 ± 0.6 b, C	93.7 ± 1.0 a, B	100 a, A	–
*L. officinalis*	18.4 ± 2.7 e, DE	19.4 ± 0.8 e, EF	28.9 ± 0.9 d, C	46.0 ± 0.4 c, CD	67.1 ± 5.7 b, C	97.1 ± 1.3 a, B	100 a, A	–
*L. hybrida*	11.8 ± 0.8 e, DE	15.9 ± 1.2 e, F	27.6 ± 2.5 d, C	45.8 ± 2.9 c, CD	77.9 ± 3.4 b, B	97.8 ± 0.7 a, B	100 a, A	–
*C. bergamia*	9.2 ± 0.4 d, DE	14.6 ± 1.2 ed, F	18.9 ± 3.5 e, D	32.5 ± 6.7 d, D	44.0 ± 2.0 c, D	75.7 ± 1.5 b, D	97.6 ± 1.1 a, A	100 a
*R. officinalis*	8.4 ± 6.5 d, DE	6.9 ± 5.9 d, FG	11.8 ± 0.8 d, E	27.6 ± 3.8 c, D	32.7 ± 2.7 c, E	67.1 ± 7.7 b, E	91.2 ± 5.1 a, B	100 a
*C. zeylanicum*	3.0 ± 1.0 c, E	-1.6 ± 1.8 c, G	20.6 ± 3.1 c, D	72.1 ± 21.8 b, B	99.0 ± 1.7 a, A	100 a, A	–	–
MIX	53.8 ± 6.7 b, C	66.1 ± 12.3 a, C	97.0 ± 0.8 a, A	100 a, A	–	–	–	–

The results are expressed as the mean ± standard deviation, r=3. The statistical analyses were carried out using one-way analysis of variance (ANOVA), followed by Tukey’s post hoc test (p ≤ 0.05) or non-parametric Kruskal–Wallis H-test, followed by Mann-Whitney U-test (p ≤ 0.05). Different lower-case letters indicate statistical differences between concentrations of the same EO (horizontal rows). Different upper-case letters indicate statistical differences between the same concentration of different EOs (vertical columns).

**Table 5:** Percentage of Inhibition (PI, %) produced by the tested EOs in vapor phase against *Monilinia fructigena*.

Essential Oil	PI (%) exerted by EO-VOCs on *Monilinia fructigena* mycelial growth 5 days post inoculation. EO concentrations are expressed in μL/L							
	2.82	5.64	11.36	22.73	45.45	90.91	181.82	363.64
*O. vulgare* 1	95.8 ± 1.5 a, AB	96.3 ± 6.4 a, A	100 a, A	–	–	–	–	–
*O. vulgare* 2	93.1 ± 3.0 b, AB	100 a, A	100 a, A	–				
*T. vulgaris* 1	66.7 ± 14.1 b,ABCDE	91.6 ± 4.1 a, A	98.4 ± 1.3 a, AB	100 a, A	–	–	–	–
*T. vulgaris* 2	97.0 ± 3.2 a, A	100 a, A	100 a, A	100 a, A	–	–	–	–
*T. serpyllum*	94.1 ± 6.8 a, AB	96.3 ± 3.4 a, A	100 a, A	–	–	–	–	–
*M. alternifolia* 1	63.4 ± 5.9 b, BCDE	64.2 ± 15.7 b, B	83.6 ± 2.0 a, BCD	87.6 ± 0.4 a, BC	96.7 ± 2.1 a, A	99.3 ± 1.3 a, A	100 a, A	–
*M. alternifolia* 2	62.7 ± 9.3 d, BCDE	61.8 ± 5.2 d, BC	70.8 ± 4.1 cd, CD	81.5 ± 3.9 bc, BC	92.7 ± 0.7 ab, B	98.5 ± 2.1 a, A	100 a, A	
*L. officinalis*	74.9 ± 3.0 cd, ABC	80.6 ± 3.4 bcd, AB	69.4 ± 14.0 d, D	83.1 ± 9.4 abcd, BC	90.6 ± 2.4 abc, BC	99.0 ± 0.4 ab, A	100 a, A	–
*L. hybrida*	53.7 ± 21.0 d, CDE	76.4 ± 6.3 bc, AB	86.1 ± 0.4 ab, ABC	91.8 ± 0.7 ab, AB	92.4 ± 1.5 ab, BC	100 a, A	100 a, A	–
*C. bergamia*	34.1 ± 14.9 b, EF	30.8 ± 12.8 b, D	51.7 ± 8.7 b, E	77.9 ± 4.3 a, C	89.0 ± 0.4 a, C	82.3 ± 3.9 a, B	96.1 ± 1.1 a, B	100 a
*R. officinalis*	37.6 ± 19.4 d, DEF	39.3 ± 14.6 cd, CD	32.1 ± 6.4 cd, F	61.7 ± 4.4 bc, D	80.3 ± 2.1 ab, D	85.5 ± 6.5 ab, B	96.1 ± 2.1 a, B	100 a
*C. zeylanicum*	10.1 ± 8.4 c, F	16.8 ± 3.9 c, D	12.6 ± 5.6 c, G	36.9 ± 4.6 b, E	100 a, A	100 a, A	–	–
MIX	69.1 ± 11.2 b, ABCD	86.1 ± 9.9 ab, AB	100 a, A	100 a, A	–	–	–	–

The results are expressed as the mean ± standard deviation, r=3. The statistical analyses were carried out using one-way analysis of variance (ANOVA), followed by Tukey’s post hoc test (p ≤ 0.05) or non-parametric Kruskal–Wallis H-test, followed by Mann-Whitney U-test (p ≤ 0.05). Different lower-case letters indicate statistical differences between concentrations of the same EO (horizontal rows). Different upper-case letters indicate statistical differences between the same concentration of different EOs (vertical columns).

**Table 6:** Percentage of Inhibition (PI, %) produced by the tested EOs in vapor phase against *Monilinia laxa*.

Essential Oil	PI (%) exerted by EO-VOCs on *Monilinia laxa* mycelial growth 5 days post inoculation. EO concentrations are expressed in μL/L							
	2.82	5.64	11.36	22.73	45.45	90.91	181.82	363.64
*O. vulgare* 1	94.4 ± 3.0 b, A	96.6 ± 0.4 ab, A	100 a, A	–	–	–	–	–
*O. vulgare* 2	85.8 ± 11.0 a, AB	93.3 ± 0.9 a, A	100 a, A					
*T. vulgaris* 1	72.0 ± 4.7 c, BC	89.0 ± 1.7 b, A	100 a, A	–	–	–	–	–
*T. vulgaris* 2	72.2 ± 3.8 c, BC	91.8 ± 2.0 b, A	100 a, A	–	–	–	–	–
*T. serpyllum*	70.5 ± 4.1 c, C	85.2 ± 1.7 b, AB	98.5 ± 1.3 a, A	100 a, A	–	–	–	–
*M. alternifolia* 1	10.8 ± 3.0 e, EF	17.4 ± 3.2 e, D	27.2 ± 2.1 d, BC	44.6 ± 0.8 c, BC	67.3 ± 4.8 b, C	93.5 ± 0.6 a, BC	100 a	–
*M. alternifolia* 2	22.2 ± 2.2 e, E	37.7 ± 15.8 de, C	27.0 ± 4.0 e, BC	57.3 ± 12.6 cd, B	76.2 ± 7.8 bc, B	92.7 ± 1.2 ab, C	100 a	–
*L. officinalis*	6.6 ± 7.9 d, F	13.2 ± 0.9 cd, D	23.5 ± 3.3 c, BCD	53.3 ± 11.9 b, BC	67.1 ± 1.1 b, C	97.0 ± 0.4 a, ABC	100 a	–
*L. hybrida*	5.0 ± 0.8 f, F	11.1 ± 2.4 e, D	16.9 ± 2.4 d, CD	39.3 ± 0.5 c, C	77.6 ± 2.6 b, B	98.5 ± 1.5 a, AB	100 a	–
*C. bergamia*	1.8 ± 3.2 e, F	3.7 ± 2.4 e, D	16.1 ± 2.8 d, CD	17.4 ± 3.6 b, D	31.1 ± 2.1 c, D	72.5 ± 5.4 b, D	100 a	100 a
*R. officinalis*	6.9 ± 4.1 e, F	8.4 ± 3.9 e, D	12.1 ± 0.8 de, D	16.6 ± 2.8 b, D	28.8 ± 1.6 c, D	58.5 ± 3.2 b, E	100 a	100 a
*C. zeylanicum*	0.0 ± 0.0 d, F	4.8 ± 6.1 d, D	31.2 ± 13.2 c, B	52.8 ± 5.4 b, BC	98.6 ± 1.1 a, A	100 a, A	–	–
MIX	55.0 ± 7.1 c, D	71.4 ± 3.9 b, B	94.8 ± 2.2 a, A	100 a, A	–	–	–	–

The results are expressed as the mean ± standard deviation, r=3. The statistical analyses were carried out using one-way analysis of variance (ANOVA), followed by Tukey’s post hoc test (p ≤ 0.05) or non-parametric Kruskal–Wallis H-test, followed by Mann-Whitney U-test (p ≤ 0.05). Different lower-case letters indicate statistical differences between concentrations of the same EO (horizontal rows). Different upper-case letters indicate statistical differences between the same concentration of different EOs (vertical columns).

*B. cinerea* (**Table 3**) was usually less affected by EO volatiles than all three *Monilinia* spp. (**Tables 4**–**6**), with differences up to more than 20% regarding the same concentration and treatment. For example, 5.64 μL/L of *O. vulgare* 1 EO showed inhibitions of 82.9% against *B. cinerea* and 100%, 96.3%, and 96.6% against *M. fructicola*, *M. fructigena* and *M. laxa*, respectively; or 22.73 μL/L of *M. alternifolia* 1 EO produced PIs of 23.6% against *B. cinerea* and 57.3% for *M. fructicola*, 87.6% for *M. fructigena*, and 44.6% for *M. laxa*. *M. fructicola* and *M. laxa* had lower PIs than *B. cinerea* when exposed to some concentrations of certain EOs, such as *M. alternifolia* 2, *L. hybrida*, *C. bergamia*, or *R. officinalis*, especially in their lower doses. *C. zeylanicum* EO was the only treatment that consistently showed higher inhibitory activity towards *B. cinerea* than to the tested *Monilinia* spp. For example, using the lowest concentration of 2.82 μL/L, the results were 0.0%, 3.0%, 1.0%, and 10.1% for *M. fructicola*, *M. fructigena*, and *M. laxa*, respectively, and 37.9% for *B. cinerea*. These differences were even higher in subsequent concentrations, with 11.36 μL/L of *C. zeylanicum* EO producing PIs of 20.6%, 12.6%, and 31.2% against *M. fructicola*, *M. fructigena*, and *M. laxa*, and of 69.9% against *B. cinerea*. In general, *M. fructigena* (**Table 5**) was the most inhibited among the three *Monilinia* spp. strains, although it presented a higher MIC value for *T. vulgaris* EO and higher than *M. laxa* for *C. bergamia* and *R. officinalis* (**Table 2**).

No fungicidal activity was observed for any of the EOs at the tested concentrations, since all fungal plugs from treatments with complete inhibition were able to grow after re-inoculation on fresh PDA, demonstrating the fungistatic effect of the EO VOCs.

## 4 Discussion

This study indicates that EO VOCs show antimicrobial activity toward *B. cinerea*, *M. fructicola*, *M. fructigena*, and *M. laxa*, and a dose-dependent inhibitory activity. *B. cinerea* usually showed a lower sensitivity to the tested EOs in comparison to the three tested *Monilinia* spp. This result confirms previous studies, in which *M. fructicola* proved to be more affected by several EO VOCs than *B. cinerea* (Santoro et al., 2018). These authors reported an increased incidence of gray mold in both nectarines and peaches treated with thyme and savory EOs, which they related to the reduction in brown rot derived from the treatment. *T. vulgaris* EO MIC on *B. cinerea* (26.7 μL/L) was reported to be double that on *M. laxa* (13.3 μL/L) (Pinto et al., 2020), which are very much in line with our results (22.73-45.45 μL/L for B. cinerea and 11.36 μL/L for *M. laxa*). These differences were even higher when testing the effects of its individual components, namely thymol and p-cymene. Conversely, in the present study, *C. zeylanicum* EO VOCs were the only ones that produced a consistently higher antifungal activity on *B. cinerea* than against any of the tested *Monilinia* spp. strains. These results could point to a different mode of action, thus further research would be of interest to understand the specific traits of this EO.

*O. vulgare*, *T. vulgaris*, and *T. serpyllum* presented the highest antifungal activity of all the tested EOs. This is again in accordance with previous research that showed their strong inhibitory effects (Paris et al., 2020; Pinto et al., 2020: Yan et al., 2021; Di Francesco et al., 2022; Elsayed et al., 2022; Kara et al., 2022; Zanotto et al., 2023). The commercial mixture of *M. alternifolia*, *O. vulgare*, *C. zeylanicum* and *T. vulgaris* presented quite high inhibitory activity, although this was lower than that of the most active of its components (*O. vulgare*), similar to that of *T. vulgaris*, but stronger than that of *M. alternifolia* and *C. zeylanicum*. These results do not indicate a synergistic activity of the listed EOs. Previous studies have reported both synergistic and non-synergistic results concerning microbial inhibition from EO mixtures (Goñi et al., 2009).

Among the four tested fungi, *B. cinerea* is the most studied one concerning the antifungal activity of vapor phase EOs, both *in vitro* and *in vivo* (Di Francesco et al., 2022; Elsayed et al., 2022). Out of the twelve applied EOs, *T. serpyllum* and *C. bergamia* seem to be the only ones that had not yet been tested against this pathogen. The strong antifungal effect of vapor-phase EOs from *O. vulgare* and other *Origanum* species on *B. cinerea* has been extensively reported (Munhuweyi et al., 2017; Zhao et al., 2021; Elsayed et al., 2022; Kaharamanoglu et al., 2022; Kara et al., 2022). Zhao et al. (2021) found significant inhibitory activity of *O. vulgare* EO VOCs on *B. cinerea* and proved that this activity was significantly higher in volatile rather than in direct contact, although the MIC they reported was considerably higher than the obtained in the present study (around 250 μg/L in comparison to 22.73 μL/L), while they reported a MIC of around 15.63 μg/L for carvacrol and thymol. We could hypothesized that these differences derive from the lower level of carvacrol in the *O. vulgare* EO used by these authors (15% in comparison to 69% and 79% in the present study). Several studies have also demonstrated the strong antifungal effect of *T. vulgaris* and *C. zeylanicum* EOs against *B. cinerea* (Paris et al., 2020; Pinto et al., 2020; Di Francesco et al., 2022; Elsayed et al., 2022), which is confirmed in our investigations. Elsayed et al. (2022) found significant disease reduction on grapes treated with these EOs, both applied with spray or fumigation. Yan et al. (2021) reported *in vitro* PIs close to 100% at 50 μL/L for *T. vulgaris* and *Origanum heracleoticum* EOs. In our case, this inhibition was reached at lower doses (22.73 to 45.45 μL/L). These same authors reported that EOs from *Melaleuca* spp., *Lavandula* spp., and *R. officinalis* were less effective against *B. cinerea*, as also found in our trials. For example, they reported a PI of 72.3%, very similar to the 78.4% we observed at 181.82 μL/L for *R. officinalis* EO. Other studies further support these findings, with *L. hybrida* showing a half maximal inhibitory concentration (IC_50_) around one order of magnitude higher that of *T. vulgaris* (Maietti et al., 2013), and a similar outcome was reported for *R. officinalis* and *Lavandula angustifolia* (Di Francesco et al., 2022). Yu et al. (2015) reported that the treatment with *M. alternifolia* EO constituents led to pronounced alterations in cellular ultrastructure, mycelial morphology, and membrane permeability, linked to a reduction in ergosterol levels.

Concerning *M. fructicola*, previous studies indicate that *T. vulgaris* EO inhibits its growth both *in vitro* and *in vivo*, being able to reduce brown rot incidence in nectarines and peaches, while promoting gray mold (Santoro et al., 2018). This was an effect that the authors related to the higher resistance of *B. cinerea* to the volatiles, which would also benefit from the niche left by the *Monilinia* sp. This same study reported that thyme and savory EOs effectively inhibit spore germination and germ tube elongation of *M. fructicola*. The inhibitory activity of *T. vulgaris* EO against *M. fructicola* has been further reported by other studies (Sellamuthu et al., 2013; Spadaro et al., 2021). *M. alternifolia* EO has been tested in vapor phase against *M. fructicola* (Xu et al., 2022). These authors used an EO-liposome formulation, obtaining PIs at least one order of magnitude lower than those from our investigation. They ascribed this low inhibitory activity to the slow release of volatiles from the liposomes (Xu et al., 2022). Xiong et al. (2021) reported a relevant inhibitory activity of lavender EO on *M. fructicola*, both in direct contact and with fumigation. These authors associated the antifungal effects of *Lavandula* sp. EO to cytoplasm leakage, hyphal and spore distortion, and cell membrane damage. They also reported an increased expression of apoptosis related genes in the exposed colonies. As far as we know, no previous vapor phase studies have been conducted with *O. vulgare*, *T. serpyllum*, *C. bergamia*, *R. officinalis*, or *C. zeylanicum* EO VOCs on *M. fructicola*, although some of them have been tested in direct contact (Lazar-Baker et al., 2011; Grulová et al., 2020; Xu et al., 2021). In this regard, Xu et al. (2021) reported that EOs such as *T. vulgaris* and *M. alternifolia* affect the structure of *M. fructicola* cell membrane, leading to changes in mycelial morphology, membrane permeability, and levels of intracellular reactive oxygen species.

The reported general higher PIs for *M. fructigena* in comparison to the other fungi suggest a higher sensitivity to most vapor-phase EOs than that shown by *B. cinerea* and the other tested *Monilinia* spp. These results cannot be compared with previous work, since we have found no published research assessing the effect of EOs in the vapor phase against *M. fructigena*. However, there are some studies concerning the antifungal activity of some EOs on this pathogen using the agar dilution method. El Khetabi et al. (2021) found small differences between *M. fructigena* and *M. laxa*, reporting similar growth inhibition for both fungi exposed to several EOs, including *T. vulgaris*, *R. officinalis*, and other species from the genus *Origanum*, *Lavandula*, and *Citrus*. Elshafie et al. (2015a; 2015b) also reported the important inhibitory activity of *O. vulgare* and *T. vulgaris* EOs and some of their constituents, such as carvacrol and thymol, against *M. fructicola*, *M. fructigena*, and *M. laxa* both *in vitro* and *in vivo*, although they did not highlight relevant differences among these fungal species.

With regard to *M. laxa*, as far as we know, out of the twelve individual EOs tested in our investigation, only *T. vulgaris* and *C. zeylanicum* had already been assayed in vapor phase against this pathogen. Pinto et al. (2020) observed a strong *in vitro* inhibitory activity of *T. vulgaris* EO VOCs and its individual components thymol, p-cymene, and ɣ-terpinene on *M. laxa*. These authors reported an MIC of 13.3 μL/L, which is similar to the one we obtained (11.36 μL/L). *In vivo* assays indicate that *T. vulgaris* and *C. zeylanicum* EO VOCs can also protect peaches from brown rot caused by *M. laxa* via the induction of physiological and defensive responses in the fruit (Cindi et al., 2016; Khumalo et al., 2017). EO VOCs from different *Ocimum basilicum* varieties also demonstrated *in vitro* antifungal activity against this pathogen (Carović-Stanco et al., 2013).

When considering their major components, our results suggest that EOs with significant levels of carvacrol and thymol (namely *O. vulgare*, *T. vulgaris*, and *T. serpyllum*) show higher antifungal activities than the others. This is supported by previous reports, in which these individual compounds demonstrated strong antifungal activity (Elshafie et al., 2015b; Pinto et al., 2020; Buonsenso et al., 2023), even presenting in some cases a MIC one order of magnitude lower than the whole essential oil, for example thymol MIC against *M. laxa* was 1.6 μL/L, while thyme EO MIC was 13.3 μL/L (Pinto et al., 2020). Kara et al. (2022) have also highlighted the inhibitory effects of EOs rich in carvacrol and thymol.

As far as we know, this is the first report in which VOC chambers have been used to evaluate the antimicrobial effects of volatile compounds from plant EOs. They have proven to be an effective alternative methodology for the study of these interactions. This protocol could be especially useful for a second phase of *in vitro* experiments after using a simpler screening method (Kloucek et al., 2012; Cernava et al., 2015), or as a transition for *in vivo* or *ex vivo* trials (Álvarez-García et al., 2022a).

## 5 Conclusions

VOC chambers proved to be an effective method to evaluate the antimicrobial activity of volatile EOs. The tested EOs presented significant concentration-dependent antifungal activity against *B. cinerea*, *M. fructicola*, *M. fructigena*, and *M. laxa*. Overall, *B. cinerea* was less inhibited than the three *Monilinia* spp., except for *C. zeylanicum* EO, which consistently showed higher inhibition against *B. cinerea*, with a MIC of 45.45 μL/L in comparison to 90.91 μL/L against *M. laxa* and *M. fructicola*. Among the *Monilinia* strains, *M. fructigena* was the most sensitive, followed by *M. laxa*, and being *M. fructicola* the most resistant one.

*O. vulgare*, *T. vulgaris*, and *T. serpyllum* EOs volatiles presented the highest inhibitory activity, with a MIC of 22.73, 45.45, and 22.73 µL/L, respectively, against *B. cinerea* and a range between 5.64 and 22.73 μL/L against the three *Monilinia* spp. *M. alternifolia*, *L. officinalis* and *L. hybrida* EOs showed intermediate antifungal activity, with MICs ranging from 181.82 to 363.64 μL/L against *B. cinerea* and from 90.91 to 181.82 in the case of *Monilinia* spp. *C. bergamia* and *R. officinalis* EOs were the least effective ones, with a MIC of 363.64 μL/L against *B. cinerea*, *M. fructicola*, and *M. fructigena*; and 181.82 μL/L against *M. laxa*. Further *in vivo* assays should be conducted to elucidate whether some of these EOs could be of use for the postharvest control of gray mold and brown rot infections in fruits and vegetables.

## Data availability statement

The raw data supporting the conclusions of this article will be made available by the authors, without undue reservation.

## Material availability statement

VOC Chambers will be made available to researchers upon reasonable request, unless commercial agreements reached with third parties regarding the patent exploitation prohibit it (in which case the VOC Chambers should be available in the market).

## Author contributions

SÁ-G: Conceptualization, Validation, Data curation, Formal Analysis, Investigation, Methodology, Software, Visualization, Writing – original draft. MM: Formal Analysis, Investigation, Software, Validation, Writing – review & editing. GR: Validation, Writing – review & editing, Conceptualization, Funding acquisition, Project administration, Resources, Supervision.
